# Effect of experimental hypoosmolar hyponatremia on the blood brain barrier and brain edema formation

**DOI:** 10.1038/s41598-025-06320-2

**Published:** 2025-07-02

**Authors:** Marta Aleksandrowicz, Przemysław Wencel, Mateusz Kciuk, Mariusz Popek, Łukasz Przykaza

**Affiliations:** 1 Laboratory of Preclinical Research and Environmental Agents, Mossakowski Medical Research Institute, Polish Academy of Sciences, A. Pawińskiego Str. 5, 02-106 Warsaw, Poland; 2 Department of Neurotoxicology, Mossakowski Medical Research Institute, Polish Academy of Sciences, Warsaw, Poland; 3 Department of Molecular Biotechnology and Genetics, Faculty of Biology and Environmental Protection, University of Lodz, 90-237 Lodz, Poland

**Keywords:** BBB, Vasogenic edema, Cytotoxic edema, Tight junctions, Hyponatremia, Vasopressin

## Abstract

The most frequent cause of hyponatremia is water retention due to the increase in blood levels of antidiuretic hormone (vasopressin, AVP). Hyponatremia is associated with neurological deficits, which are mainly linked to brain edema. Although the cytotoxic mechanism of brain edema in hyponatremia is commonly known, the role of a vasogenic mechanism is ambiguous. The present studies aimed to evaluate the effect of both acute and chronic hyponatremia on brain edema, blood–brain barrier (BBB) permeability, and mRNA expression of the tight junctions. Acute hyponatremia was induced for 5 h by subcutaneous (s.c.) injection of vasopressin or a synthetic analog of vasopressin-desmopressin (dDAVP) with intraperitoneal (i.p.) water loading in the amount of 11% body weight. Chronic hyponatremia was induced for 4 days with the help of AVP or dDAVP released continuously from subcutaneously implanted ALZET mini-osmotic pumps and a liquid diet. Because of the vascular action of vasopressin, either vasopressin or desmopressin was used to induce hyponatremia to assess whether the observed changes were characteristic of AVP-associated hyponatremia or hyponatremia alone. Brain water content was determined using the wet-dry method. BBB permeability was studied using sodium fluorescein. Gene expression of claudin-5, occludin, and zonula occludens (ZO-1) was assessed by real-time PCR (RT-PCR). Osmolarity, Na^+^, and Cl^−^ concentrations were analyzed using the electrolyte and blood gas analyzer. Hypoosmotic acute hyponatremia led to increased brain water content and downregulation of tight junction gene expression, although leakage of the BBB was not observed. These results, except for the gene expression of claudin-5, were comparable in both groups with acute hyponatremia, regardless of whether AVP or dDAVP induced it. In chronic hyponatremia, irrespective of the mode of induction, there were no changes in the studied parameters. These results demonstrate a new insight into the nature of edema in acute hypoosmotic hyponatremia due to signs of vasogenic edema.

**Supplementary Information:**

The online version contains supplementary material available at 10.1038/s41598-025-06320-2.

## Introduction

Hyponatremia is the most common water-electrolyte balance disorder. It is considered to be an independent predictor of death and disability in patients with cancer, heart failure, liver cirrhosis, and adrenal insufficiency^1–3^. However, it exerts the most adverse effect on patients with neurological disorders, such as subarachnoid haemorrhage and traumatic brain injury^4–7^. The onset of hyponatremia can be rapid (plasma sodium concentration decreases to below 135 mM in less than 48 h) or slow (plasma sodium concentration decreases below 135 mM in more than 48 h)^8,9^. These two types of hyponatremia are named acute and chronic, respectively. Clinical symptoms such as headache, seizures, and confusion are associated with the central nervous system, primarily with hypoosmotic brain swelling. They are more evident when the decrease in the serum sodium concentration is significant or fast, i.e., in acute hyponatremia^5,9–12^. In slowly developing chronic hyponatremia, the mechanism of a “regulatory volume decrease” (RVD) enables an effective reduction of brain water due to the removal of osmotically active compounds from the cells, allowing for the cerebral symptoms to be less pronounced^9,13,14^. Regardless of this state of knowledge, many unknown mechanisms are still involved in the influence of hyponatremia on brain function, such as the presence of cognitive impairments in chronic hyponatremia^15–19^, despite developed adaptive mechanisms.

In most cases, hyponatremia arises secondary to the increase in serum concentration of antidiuretic hormone (vasopressin, AVP)^6,8,20,21^. However, in the vast majority of experimental studies, hyponatremia was induced with a vasopressin analog, desmopressin (dDAVP)^13,15,17,22–28^, which acts on renal V_2_ receptors, leading to antidiuresis. In contrast to desmopressin, vasopressin exerts vascular action, which may complicate the interpretation of the study results^13,28^. Vasopressin constricts blood vessels by acting on smooth muscle V_1_ receptors^29^ or may cause nitric oxide (NO)-dependent vasodilation by activating V_1_ receptors in endothelial cells^30^. Our previous studies have shown that in the in vitro model of acute AVP-associated hyponatremia, constriction, and disturbed endothelium function in rat’s intracerebral parenchymal arterioles are present^31^. Endothelial cells in the cerebral microvessels form the blood–brain barrier (BBB), which limits the transportation to the brain of unwanted substances circulating in the blood. Cerebral endothelial cells are connected by tight junctions (TJ), composed of integral membrane proteins such as occludin and claudin-5. Both membrane proteins are linked to the actin cytoskeleton by TJ-associated proteins such as zonula occludens-1 (ZO-1)^32^. TJ proteins claudin and occludin are heterogeneously expressed in endothelial cells of brain microvessels^33^. The adverse effect of hyponatremia associated with vasopressin on the endothelium may shed new light on the function of the BBB and the nature of edema formation. Although the cytotoxic mechanism of brain edema in hyponatremia is commonly known^8,9,34–36^, the role of a vasogenic mechanism is ambiguous. The present studies aimed to evaluate the effect of both acute and chronic hyponatremia on brain edema, BBB permeability, and tight junction mRNA expression. To assess whether the examined mechanisms are characteristic of hyponatremia alone or hyponatremia associated with vasopressin, hyponatremia was induced by vasopressin or desmopressin.

## Methods

### Animals

Male Wistar rats (body weight 270–330 g, n = 110 animals) were supplied by the Animal House of the Mossakowski Medical Research Centre, Warsaw, Poland. All animal experiments were performed in accordance with the Law and Regulations on Animal Protection in Poland (Dz.U. 2015/266) and were approved by the Extramural Second Committee for the Care and Use of Laboratory Animals for Experimental Procedures, National Medicines Institute in Warsaw (WAW2/104/23). All procedures were performed in accordance with the ARRIVE guidelines.

Animals were housed in a standard animal facility under constant temperature (23 °C) and a 12-h/12-h light/dark cycle. Rats had ad libitum access to standard chow and tap water until induction of hyponatremia. In order to withdraw brains, all animals were euthanized by decapitation with a guillotine.

### Induction of acute and chronic hypoosmotic hyponatremia

**Fig. 1:**
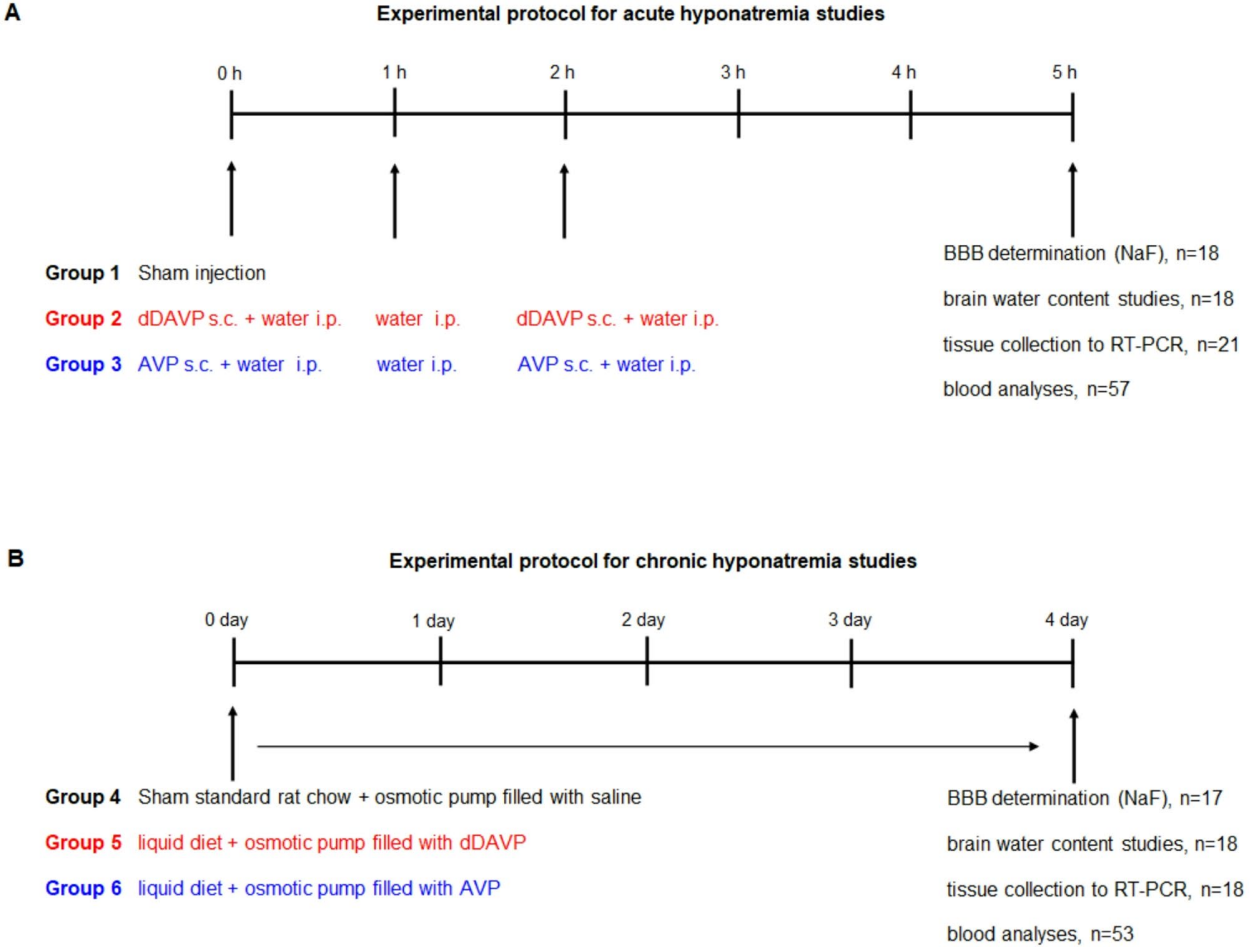
(**A**) Experimental protocol for acute hyponatremia. Acute hyponatremia was induced for 5 h by s.c. injection of AVP (1 μg) or dDAVP (0.4 μg) with i.p. water loading in the amount of 11% body weight, (**B**) Experimental protocol for chronic hyponatremia. Chronic hyponatremia was induced with AVP (2.4 μg/24 h) or dDAVP (0.12 μg/24 h) released continuously from subcutaneously implanted ALZET mini-osmotic pumps and a liquid diet. Chronic hyponatremia was maintained for 4 days.

Acute hyponatremia was induced for 5 h by the intraperitoneal injection (i.p.) of water in the amount of 11% body weight divided into 3 doses administered at 0, 1, and 2 h. Simultaneously, animals received subcutaneously (s.c.) vasopressin (AVP, 1 μg/200 μl saline, Sigma-Aldrich/Merck) or vasopressin V_2_ receptor agonist, 1-deamino-8-d-arginine vasopressin (dDAVP, 0.4 μg/200 μl saline, Sigma-Aldrich/Merck) divided into two doses at 0 and 2 h^ 23,37,38^. Sham-injected rats served as controls (Fig. 1A).

Chronic hyponatremia was induced within 4 days using an AVP- or dDAVP-filled ALZET mini-osmotic pump (Model 2002, Durect Corp., Cupertino, USA) and a rodent liquid diet (AIN-76, Bio-Serv, NJ, USA)^39^. First, the rats were acclimated for 2 days to a rodent liquid diet administered in the amount of 75 ml (0.32 g/ml). During this time, they had free access to drinking water. Osmotic pumps filled with AVP or dDAVP were implanted subcutaneously on the neck under 90 mg/kg ketamine and 10 mg/kg xylazine anesthesia. The pumps release vasopressin at a rate of 2.4 μg/24 h and desmopressin at a rate of 0.12 μg/24 h. From the second day after pump implantation, the rats were given 50 ml of the more concentrated diet (0.54 g/ml)^24^. At this time, drinking water was withheld. Sham-operated rats were implanted with osmotic pumps filled with physiological saline and had free access to drinking water and standard rat chow (Fig. 1B).

### Determination of the BBB permeability by sodium fluorescein

The experiments were performed under ketamine (90 mg/kg) and xylazine (10 mg/kg) anesthesia. The BBB permeability was assessed using a small molecular weight dye, sodium fluorescein (NaF, 376 Da, Sigma-Aldrich/Merck). Sodium fluorescein (50 mg/mL saline) was injected via the cannulated femoral vein at 0.2 mL/100 g. The marker was allowed to circulate in the blood for 30 min. Blood was withdrawn to assess NaF concentration in plasma. Next, the rats were transcardially perfused with 300 ml of ice-cold phosphate-buffered saline (PBS). The brains were removed from the scull, the brainstem and the cerebellum were discarded, and the remaining part of the brain was weighed, frozen on dry ice, and stored at − 80 °C. To determine the permeability, tissue was homogenized 1:10 (w/v) in sterile PBS. Samples were precipitated with ethanol (1:3 v/v) and centrifuged at 3000 × g for 10 min. The supernatants were diluted and analyzed in a spectrofluorometer, FLUOstar Omega (BMG Labtech, Ortenberg, Germany), using an excitation wavelength of 480 nm and an emission wavelength of 538 nm. The content of NaF in plasma and brain tissue samples was calculated from the calibration curve. The degree of the BBB permeability was measured as the ratio of NaF in a gram of brain tissue per the amount of NaF in a milliliter of plasma^40^. The brain and serum fluorescence comparison aimed to standardize the level of the BBB permeability found in various animals.

### Gene expression analysis

The brain cortex was isolated on ice and flash-frozen. RNA was isolated using the Total RNA Mini Kit (A&A Biotechnology, Poland) according to the manufacturer’s instructions. DNA was digested with DNase I (Sigma-Aldrich/Merck). The concentration and purity of RNA were assessed spectrophotometrically (A260/A280 method). Reverse transcription of 2 μg of total RNA was performed with the High-Capacity Reverse Transcription Kit (Applied Biosystems). Then, real-time PCR was performed with TaqMan Gene Expression Assay kits on the Applied Biosystems 7500 Real-Time PCR System using specific rat primers: Cldn5 (Rn01753146_s1), Ocln (Rn00580064_m1), and Tjp1 (Rn07315717_m1). Each sample was analyzed in tri- or quadruplicates. The relative mRNA expression levels [RQ] were calculated using the ΔΔCt method and normalized against beta-actin and peptidylprolyl isomerase A (cyclophilin A) (Actb: Rn00667869_m1 and Ppia: Rn00690933_m1, respectively).

### Brain water content

Brain water content was calculated using the wet-dry method. Brains were removed and weighed (wet weight). Samples were placed in an oven at 100 °C for 24 h and again weighed (dry weight). The percentage of water in each sample was calculated as follows: percent brain water = [(wet weight − dry weight)/wet weight] × 100.

### Measurements of electrolyte concentration and osmolarity

To assess the plasma osmolarity, Na^+^ and Cl^−^ concentration, blood was collected during decapitation or from the cannulated femoral artery into a 100 μl heparin-coated capillary and analyzed using the electrolyte and blood gas analyzer (Edan i5).

### Evaluation of neurological manifestations and mortality

Neurological scores in acute and chronic hyponatremia were assigned according to the following modified criteria reported previously by Sugimura et al.^41^: 6-no neurological manifestations; 5-slow or awkward gait; 4-limb weakness and/or paralysis; 3-seizures; 2-severe motor deficits; 1-complete inability to move; and 0-death.

### Data analysis and statistics

Statistical analyses were performed using Statistica software. The charts were prepared using GraphPad Prism software. The results are expressed as means ± S.E.M. The data were analyzed using one-way ANOVA followed by Tukey’s multiple comparison tests. Differences with *p* < 0.05 were considered statistically significant.

## Results

### Plasma electrolytes and osmolarity

As shown in Table 1, the combined administration of water and AVP or dDAVP induced a progressive fall in plasma sodium and chloride concentration (*p* < 0.001). Plasma osmolarity fell in a manner corresponding to the decrease in electrolytes. Similarly, in chronic hyponatremia induced by AVP or dDAVP, the concentration of sodium, chloride, and osmolarity decreased (*p* < 0.001). There were no statistically significant differences in the tested parameters depending on the duration of hyponatremia or the method of induction (AVP vs. dDAVP). In studies determining the BBB permeability, the presence of sodium fluorescein in circulatory system slightly increased plasma sodium concentration by 2 mM ± 1 (n = 5, *p* < 0.05).

**Table 1:** Plasma osmolarity and sodium and chloride concentration for each group.

	Acute hyponatremia			Chronic hyponatremia		
	Sham	dDAVP	AVP	Sham	dDAVP	AVP
sodium (mmol/L)	138 ± 0.4	113 ± 0.7	116 ± 0.7	137 ± 0.5	107 ± 1.0	112 ± 1.6
chloride (mmol/L)	102 ± 0.6	80 ± 0.9	80 ± 1.0	100 ± 0.6	73 ± 1.2	75 ± 1.5
osmolarity (mOsm/L)	287 ± 1.9	242 ± 1.9	246 ± 1.4	287 ± 0.6	231 ± 1.6	240 ± 3.2
n	19	18	20	17	18	18

Data are presented as the mean ± S.E.M

### Brain water content

**Fig. 2:**
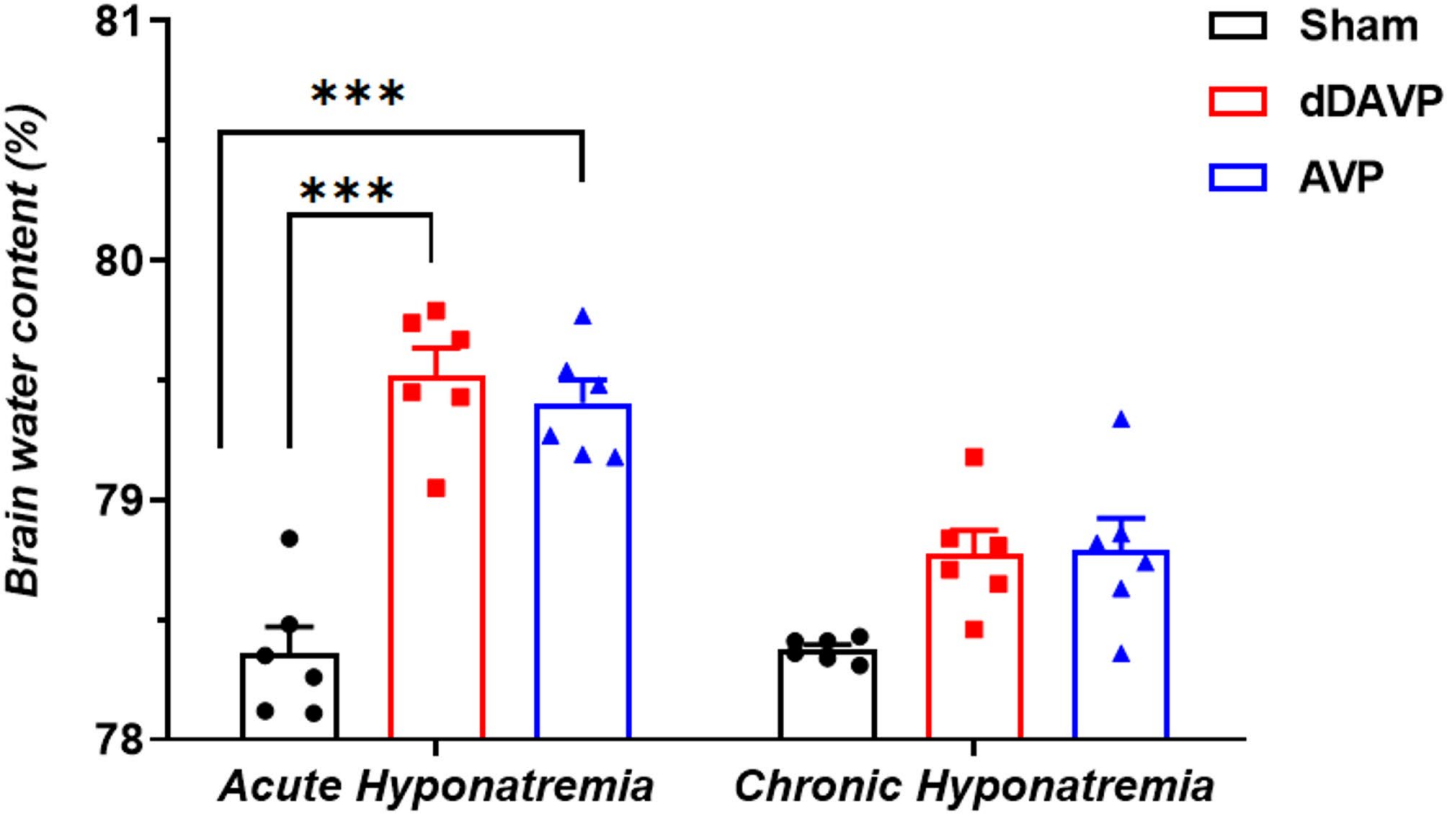
Wet–dry analysis of brain water content in AVP- and dDAVP-induced acute and chronic hyponatremia. Values are expressed as mean ± S.E.M. ***Significantly different from the sham-operated group (*p* < 0.001).

Brain water content increased in acute AVP- and dDAVP-induced hyponatremia (respectively to 79.52% ± 0.11, n = 6 and to 79.40% ± 0.09, n = 6 vs. 78.36% ± 0.11 in Sham, n = 6, *p* < 0.001). There were no differences between either group with acute hyponatremia (*p* = 0.96). In contrast, in chronic groups, no increased brain water content was observed neither in AVP- (*p* = 0.053, n = 6) nor dDAVP-induced hyponatremia (*p* = 0.068, n = 6 vs. Sham group, n = 6). These results indicate that in chronic hyponatremia lasting 4 days, the brain water content returned to normal levels (Fig. 2).

### BBB permeability

**Fig. 3:**
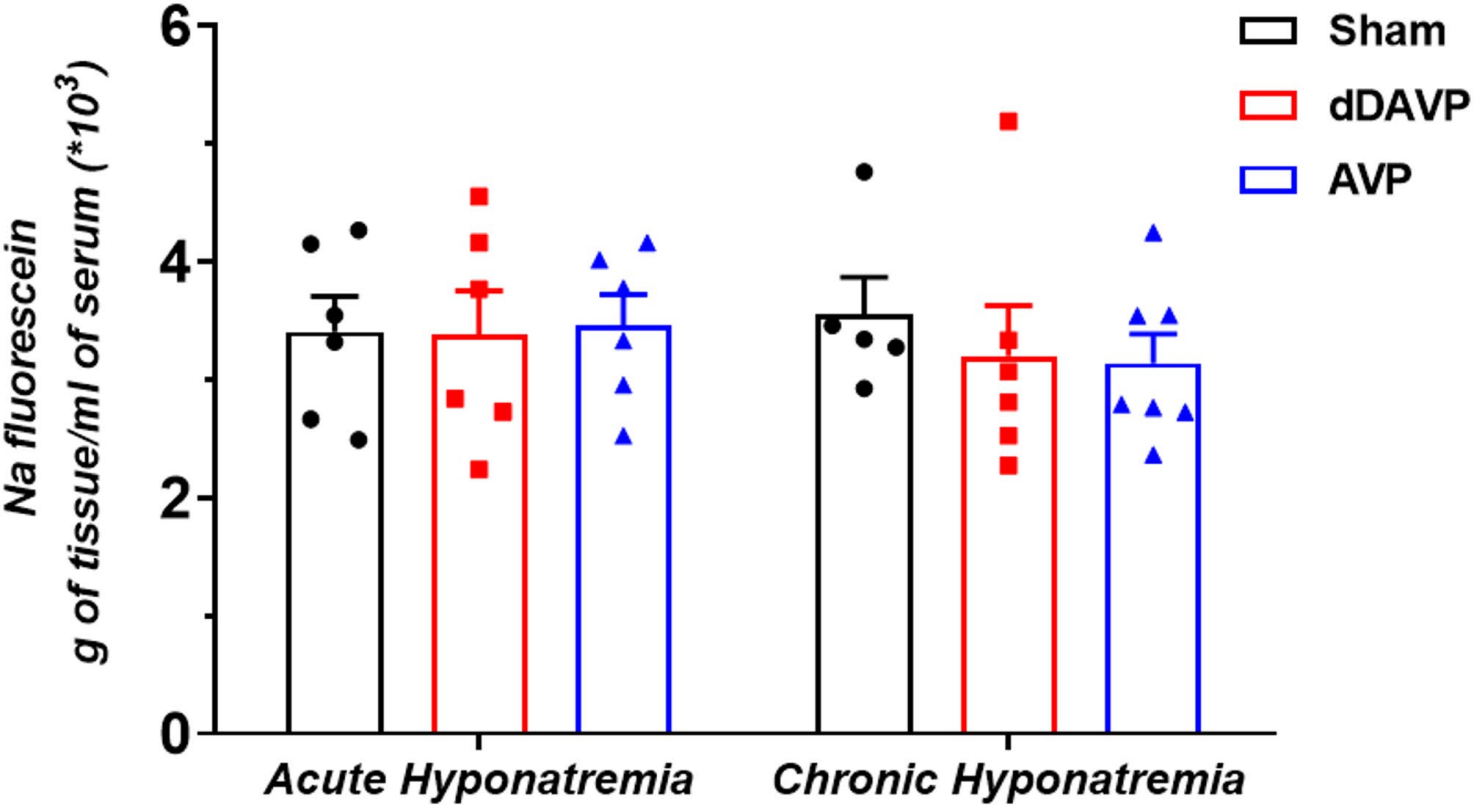
Sodium fluorescein extravasation in brains (without brainstem and the cerebellum) of rats subjected to AVP- or dDAVP-induced acute and chronic hyponatremia. Values are expressed as mean ± S.E.M.

To evaluate whether acute and chronic hyponatremia alters the permeability of the BBB, a low-weight sodium fluorescein was used. We did not observe any differences in the brain concentration of sodium fluorescein between acute AVP- (*p* = 0.99, n = 6) or dDAVP-induced hyponatremia (*p* = 0.99, n = 6) vs. Sham group (n = 6). Similar results were obtained in the groups with chronic hyponatremia, i.e., there were no differences between AVP-induced chronic hyponatremia (*p* = 0.96, n = 6) or dDAVP-induced chronic hyponatremia (*p* = 0.98, n = 6), vs. Sham group (n = 5). It means that hyponatremia, regardless of its duration (acute vs. chronic) or mode of induction (AVP vs. dDAVP), does not affect the BBB permeability (Fig. 3).

### Gene expression

**Fig. 4:**
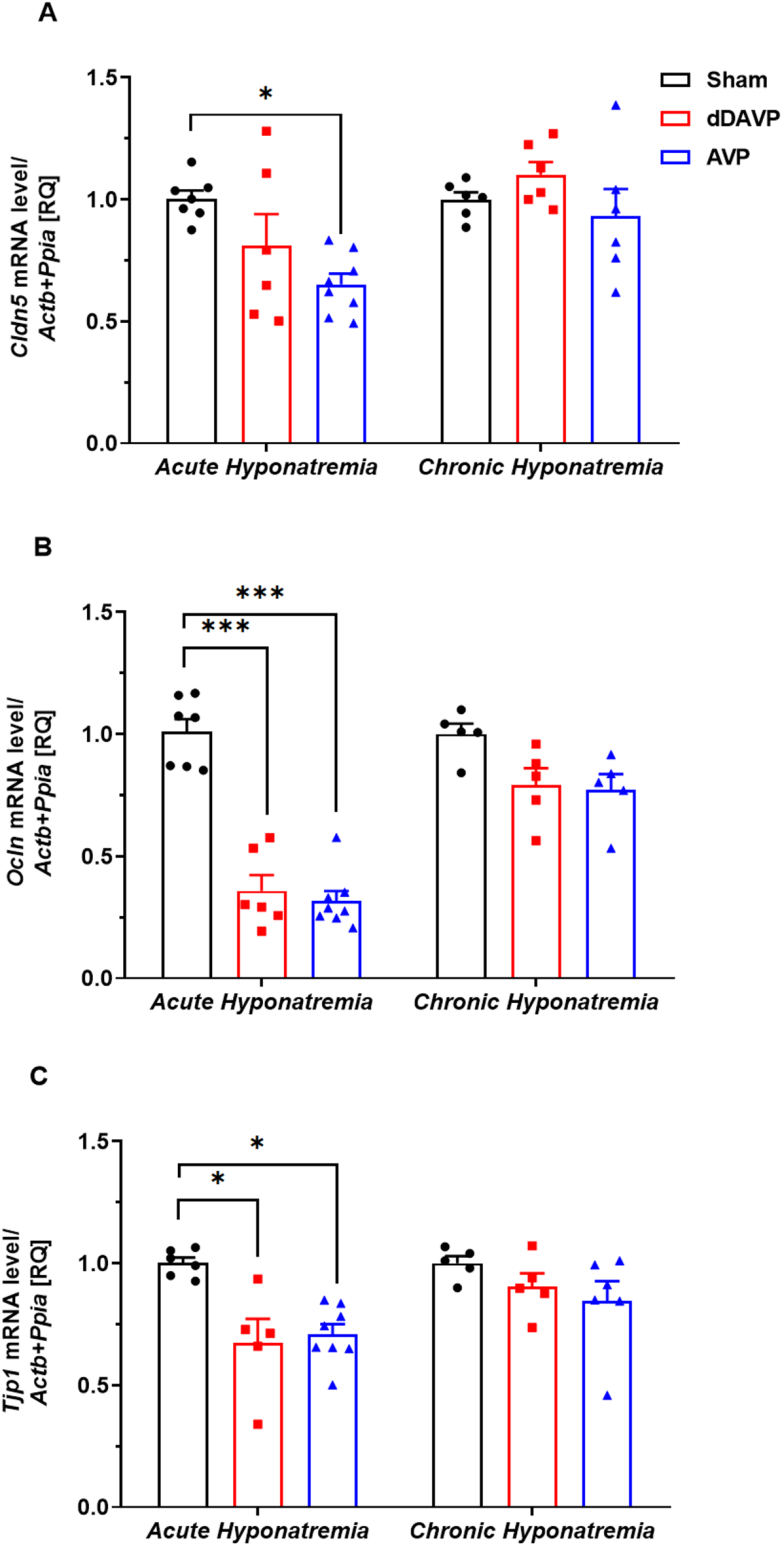
(**A**) Claudin-5, (**B**) Occludin, and (**C**) Zonula occludens-1 mRNA expression in the cortex of rats subjected to AVP- or dDAVP-induced acute and chronic hyponatremia, assessed by real-time PCR. Values are expressed as mean ± S.E.M. *p < 0.05, ***p < 0.001 vs.  Sham-operated group.

Real-time PCR was used to measure mRNA expression levels for claudin-5, occludin, and ZO-1 in the cortex in acute and chronic hyponatremia—induced by either vasopressin or desmopressin. All changes in gene expression were observed in acute but not chronic hyponatremia. There was a significant downregulation of claudin-5 mRNA in AVP-induced acute hyponatremia (*p* < 0.05, n = 8) (Fig. 4A). Levels of occludin mRNA were significantly reduced in acute hyponatremia-induced by both AVP (*p* < 0.001, n = 8) and dDAVP (*p* < 0.001, n = 6), vs. Sham group (n = 7) (Fig. 4B). Similarly, downregulation of ZO-1 mRNA was observed in acute hyponatremia-induced by both AVP (*p* < 0.05, n = 8) and dDAVP (*p* < 0.05, n = 5), vs. Sham group (n = 6) (Fig. 4C).

### Signs of abnormal central nervous system function

No mortality of rats was recorded during the entire study, both in acute and chronic hyponatremia induced by AVP and dDAVP. All animals in the chronic hyponatremia group induced by vasopressin (n = 20) or desmopressin (n = 18) appeared alert and normal. Their behavior did not differ from that of the Sham group animals. However, all animals in the groups with acute hyponatremia had slow gait and limb weakness. All animals were lethargic, and they had bristly fur. No overt coma or seizures were noted. There were no noticeable differences in behavior between AVP- or dDAVP-induced acute hyponatremia.

## Discussion

The main finding of this study is that hypoosmotic acute AVP-induced hyponatremia leads to the downregulation of tight junctions and ZO-1 gene expression, although the leakage of BBB assessed by sodium fluorescein was not observed. Increased brain water content was demonstrated in acute hyponatremia, regardless of whether it was induced by AVP or dDAVP. These observations in acute hyponatremia matched the lethargic behavior of the animals in these groups. In hypoosmolar chronic hyponatremia, we did not observe increased brain water content, changes in the expression of tight junctions, and BBB permeability.

The present studies were performed in the model of acute (5 h) and chronic (4 days) hypoosmolar hyponatremia induced by vasopressin or desmopressin. The literature review shows that induction of hyponatremia with vasopressin is rare^37–39^ compared to its induction with desmopressin^13,15,17,22–28^. The use of desmopressin was justified because it avoids the potential confounding vascular effects associated with the administration of AVP or that clinical hyponatremia was generally associated with plasma AVP concentrations below the threshold for significant vasopressor activity^13^. Increased concentration of AVP may also cause weight loss, which may contribute to higher mortality rates^13^. However, in our study, animals during the induction of chronic hyponatremia with vasopressin did not lose weight or die.

Moreover, it appears that the use of vasopressin to induce hyponatremia creates conditions that mimic pathological states associated with a decrease in sodium and an increase in vasopressin concentration in the blood, e.g., in the syndrome of inappropriate antidiuretic hormone secretion (SIADH). Induction of chronic hyponatremia using osmotic pumps, which release vasopressin at a rate of 2.4 µg/24 h, increases vasopressin concentration in the blood to 15 pg/ml^39^. This infusion rate was selected to increase AVP levels in plasma similar to the pathophysiologic concentration measured in patients with SIADH^42,43^. In vitro studies showed that vasopressin at a concentration of 15 pg/ml caused dilation of intracerebral parenchymal arterioles, but more importantly, the combination of vasopressin at this dose with a low sodium concentration (120 mM) led to arteriolar constriction and endothelial dysfunction^31^. Considering cerebral microcirculation is a critical element of proper brain functions due to the delivery of oxygen and nutrients^44^, it seems reasonable to use vasopressin to induce hyponatremia. However, to verify whether the observed results were the effect of hyponatremia combined with vasopressin or hyponatremia alone, we also used a model of hyponatremia induced with desmopressin.

Acute hyponatremia is associated with neurological symptoms such as seizures, lethargy, confusion, and gait disturbance, particularly if its onset is rapid, i.e., during acute hyponatremia^5,8,12,20,45^. The present studies have shown that animals with acute hyponatremia induced by both vasopressin and desmopressin were lethargic and were characterized by impaired mobility and limb weakness. Such symptoms of hyponatremia are traditionally linked to cytotoxic hypoosmotic cerebral edema^8,13,34,36^. Reduction of plasma osmolarity, manifested as dilutional hyponatremia, results in rapid water movement from the plasma into brain tissue. This addition of fluid to the brain results in tissue swelling^13,34^. Our studies showed an increased amount of water in the brains of rats with acute hypoosmotic hyponatremia induced either by AVP or dDAVP. Brain water content in rats’ brains increased in acute hyponatremia induced with AVP to 79.5% and with dDAVP to 79.4%, compared to 78.3% in the sham group. On the fourth day of chronic hyponatremia, the brain water content in the two hyponatremic groups (with AVP or dDAVP) was identical (78.7%), and did not differ from the sham normonatremic group. Our results are consistent with those of Verbalis^39^, who observed increased brain water content in acute AVP or dDAVP-induced hyponatremia but not chronic hyponatremia. Increased brain water content in acute hyponatremia induced by water intoxication was also reported by other groups^34,46,47^. The present studies demonstrated that the increase in water content in the brain of rats subjected to acute hyponatremia is small, about 1%. This is consistent with Melton et al.^35^, who showed that after 6 h of hyponatremia, the brain water content increases by only 40% of what is predicted based on ideal osmotic behavior. It indicates a significant degree of volume regulation just a few hours after the hypoosmotic state appears.

Traditionally, the two main types of brain edema are classified as vasogenic and cytotoxic . The increase in brain water content using the dry/wet weight method is comparable in both types of edemas^46^. However, other features allow us to distinguish the two types of edemas. Cytotoxic edema is characterized by intracellular accumulation of fluid and unchanged BBB permeability. In contrast, vasogenic edema is characterized by extravasation and extracellular accumulation of fluid into the cerebral parenchyma caused by dysfunction of the tight junctions and, disruption of the BBB^48^. It is assumed that in hypoosmotic hyponatremia, cytotoxic edema occurs^34–36^. The most recognizable feature of hypoosmotic hyponatremia is increased cell volume and decreased extracellular volume^34^. Brain edema in a hyponatremic state is primarily confined to astrocyte cell bodies^47^, a feature also characteristic of cytotoxic edema. Additionally, there were no changes in the BBB permeability in acute and chronic desmopressin-induced hyponatremia^13^ and in acute hyponatremia induced by water loading^35,36^. These recent studies on the BBB permeability are consistent with our results, which showed that in both acute and chronic hyponatremia induced by vasopressin or desmopressin, sodium fluorescein was not leaking into the brain. To our knowledge, these were the first studies on the effect of vasopressin-induced hyponatremia on the BBB permeability, tight junctions, and ZO-1 gene expression. In the absence of functional BBB dysfunction, a surprising result was decreased occludin, claudin-5, and zonula occluden-1 gene expression in acute AVP-induced hyponatremia. In dDAVP-induced acute hyponatremia, downregulation of occludin and ZO-1 was observed. Reduced gene expression of claudin-5 in AVP-induced hyponatremia and no change in dDAVP-induced hyponatremia suggest that low sodium had no effect on the expression of this tight junction. In contrast, the detrimental influence of AVP on claudin-5 expression was reported^49^.

 In chronic hyponatremia, in contrast to acute hyponatremia there were no changes in mRNA expression of tight junctions and ZO-1. These results suggest a vasogenic component of edema in hypoosmotic acute hyponatremia. Earlier studies reported this phenomenon, e.g., Olsen et al. observed an increase in extracellular space in white matter^47^, which is characteristic of vasogenic edema. Kozler and Pokorny showed increased BBB permeability in 24-h hyponatremia due to induced cytotoxic edema^50^. These authors hypothesized that while cytotoxic edema is characterized by intracellular accumulation of fluid, it makes the BBB more permeable with water accumulation in extracellular space^47^, which is, in turn, the main feature of vasogenic edema.

Moreover, electron microscopy studies described swollen cerebrovascular endothelial cells in the brains subjected to acute hyponatremia^51,52^ and a collapse of the capillary lumen^52^. Olsen et al. considered that swollen astrocyte foot processes may compress capillaries in the edematous brain^36^. These findings are consistent with the emerging concept that pure cytotoxic edema can initially pass into vasogenic edema^50^. Moreover, the coexistence of these two types of edemas and the transition from one to the other were also reported in other pathophysiological stages^53–55^.

In line with historical data, the changes in water quantity observed in acute hyponatremia, with the simultaneous absence of changes in the BBB permeability to sodium fluorescein, suggest the occurrence of cytotoxic edema, which was not observed in the chronic model. While cellular swelling manifests within minutes after acute central nervous system injuries, vasogenic edema, which includes the extravasation of plasma proteins, manifests hours after the initial insult^45,56^. Increased brain water content in acute hyponatremia correlated with the first noticeable signs of tight junction protein alteration at the mRNA level. Notably, the decrease in the mRNA level of Claudin-5, a cell-specific marker of endothelial cell tight junction proteins, was significantly reduced in the AVP group in acute hyponatremia. The early changes in mRNA expression of proteins associated with the BBB integrity in response to cytotoxic edema were only noticeable in the acute hyponatremia model, with no apparent functional effect. While significant gaps remain in our understanding of how specific proteins contribute to cerebral edema, the fields of cerebral edema and brain interstitial fluid dynamics were robust and productive. The next few years will yield new knowledge of how particular proteins drive edema influx, paving the way for rationally designed therapeutics that directly target critical steps in cerebral edema formation, thereby achieving what currently approved therapies do not.

Although the present studies showed unchanged permeability of the BBB in hypoosmolar acute hyponatremia, loss of expression of the genes coding for the tight junction proteins adds to a growing body of evidence that BBB is dysfunctional in hyponatremia. Specifically, it illustrates the coexistence of cytotoxic and vasogenic edema in acute hypoosmotic hyponatremia. It seems, however, that as in the case of hepatic encephalopathy^57^, its occurrence is only on a background of predominant cytotoxic injury. Nevertheless, understanding the mechanisms and clinical implications of cytotoxic and vasogenic edema is crucial for appropriate diagnosis and effective treatment.

## Electronic supplementary material

Below is the link to the electronic supplementary material.

Supplementary Material 1

## Data Availability

The datasets generated and analysed during the current study are available at the RepOD repository https://doi.org/10.18150/HMBMRX.
