# Supplementary material for: Effect of experimental hypoosmolar hyponatremia on the blood brain barrier and brain edema formation
Source: Sci Rep. 2025 Jul 2;15:23361. doi: 10.1038/s41598-025-06320-2 (PMC12222756; doi:10.1038/s41598-025-06320-2)
Supplement: Supplementary file 1 — Supplementary Material 1 [file 41598_2025_6320_MOESM1_ESM.docx]

Supplementary Information

Effect of vasopressin-induced acute and chronic hypoosmolar hyponatremia on BBB permeability, tight junction mRNA expression, and brain water content in rats

Marta Aleksandrowicz^1^, Przemysław Wencel^1^, Mateusz Kciuk^3^, Mariusz Popek^2^, Łukasz Przykaza^1^

^1^ Laboratory of Preclinical Research and Environmental Agents, Mossakowski Medical Research Institute, Polish Academy of Sciences, Warsaw, Poland

^2^ Department of Neurotoxicology, Mossakowski Medical Research Institute, Polish Academy of Sciences, Warsaw, Poland

^3^ Department of Molecular Biotechnology and Genetics, Faculty of Biology and Environmental Protection, University of Lodz, Lodz, Poland

Corresponding author: [maleksandrowicz@imdik.pan.pl](mailto:maleksandrowicz@imdik.pan.pl)

**Fig. 1** Weight of rats and concentration of Na^+^, K^+^, Cl^-^, and osmolarity in individual groups. Acute hyponatremia (Group I Sham; Group II dDAVP s.c. + water i.p.; Group III AVP s.c. + water i.p.). Chronic hyponatremia (Group IV Sham; Group V dDAVP released by osmotic pump + liquid diet; Group VI AVP released by osmotic pump + liquid diet).

**Fig. 2** Weight of brains [g] before and after oven drying (100°C, 24h) to estimate brain water content. Acute hyponatremia (Group I Sham; Group II dDAVP s.c. + water i.p.; Group III AVP s.c. + water i.p.). Chronic hyponatremia (Group IV Sham; Group V dDAVP released by osmotic pump + liquid diet; Group VI AVP released by osmotic pump + liquid diet).

|  | **weight of brains [g]** | | | | | | | | | | | |
| --- | --- | --- | --- | --- | --- | --- | --- | --- | --- | --- | --- | --- |
|  | **Group I** | | **Group II** | | **Group III** | | **Group IV** | | **Group V** | | **Group VI** | |
| No. | before | after | before | after | before | after | before | after | before | after | before | after |
| 1 | 1.9420 | 0.4220 | 2.0278 | 0.4248 | 1.8719 | 0.3829 | 1.9815 | 0.4279 | 2.0112 | 0.4328 | 2.0910 | 0.4318 |
| 2 | 1.9138 | 0.4048 | 2.0090 | 0.4058 | 2,1100 | 0,4267 | 1.9236 | 0.4161 | 2.0327 | 0.4339 | 1.9676 | 0.4157 |
| 3 | 1.9401 | 0.4175 | 2.0731 | 0.4199 | 1.9900 | 0.4125 | 1.9565 | 0.4220 | 1.9574 | 0.4075 | 1.8925 | 0.3999 |
| 4 | 1.9384 | 0.4205 | 2.1060 | 0.4332 | 2.0006 | 0.4104 | 1.9746 | 0.4276 | 2.0253 | 0.4310 | 2.0004 | 0.4252 |
| 5 | 1.8909 | 0.4136 | 2.0342 | 0.4180 | 2.0822 | 0.4333 | 1.9755 | 0.4281 | 2.0064 | 0.4251 | 1.7367 | 0.3757 |
| 6 | 1.9936 | 0.4363 | 2.0368 | 0.4135 | 2.0319 | 0.4229 | 1.9765 | 0.4286 | 1.9875 | 0.4205 | 1.9875 | 0.4205 |

**Fig. 3** Calculation of sodium fluorescein concentration in brain homogenate and in plasma **A)** Plasma and homogenate curve, **B**) Raw data from which sodium fluorescein concentration was calculated in brain homogenate and in plasma.

Acute hyponatremia (Group I Sham; Group II dDAVP s.c. + water i.p.; Group III AVP s.c. + water i.p.). Chronic hyponatremia (Group IV Sham; Group V dDAVP released by osmotic pump + liquid diet; Group VI AVP released by osmotic pump + liquid diet).

**A)**


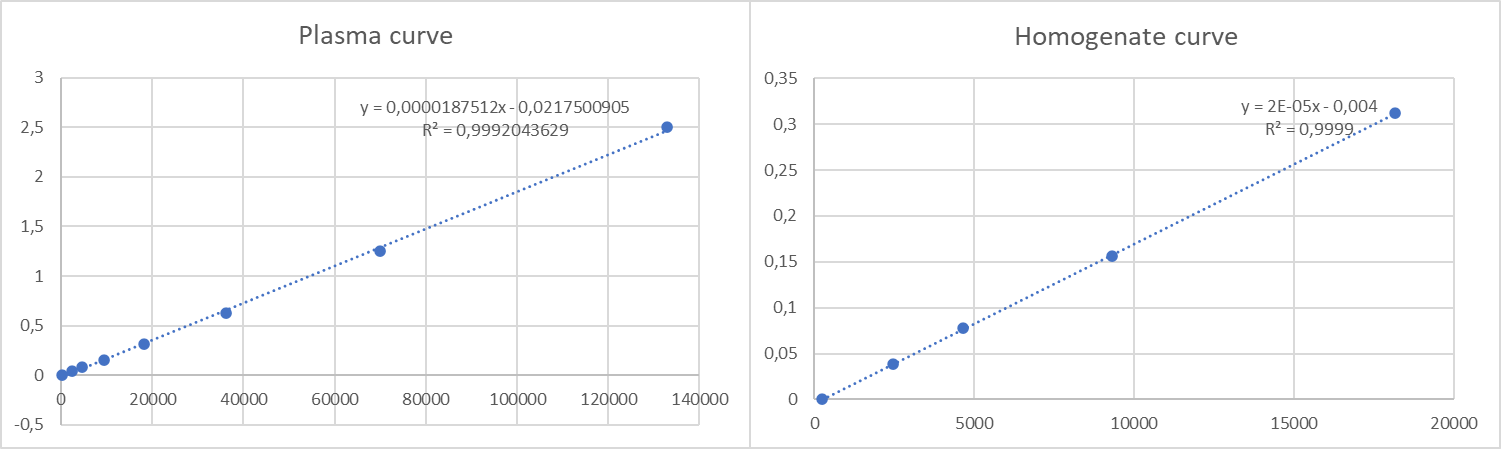


**B)**
